# Transcriptional Analysis Reveals Gender-Specific Changes in the Aging of the Human Immune System

**DOI:** 10.1371/journal.pone.0066229

**Published:** 2013-06-11

**Authors:** Saara Marttila, Juulia Jylhävä, Tapio Nevalainen, Matti Nykter, Marja Jylhä, Antti Hervonen, Liina Tserel, Pärt Peterson, Mikko Hurme

**Affiliations:** 1 Department of Microbiology and Immunology, School of Medicine, University of Tampere, Tampere, Finland; 2 Gerontology Research Center, Tampere, Finland; 3 Institute of Biomedical Technology, University of Tampere, Tampere, Finland; 4 School of Health Sciences, University of Tampere, Tampere, Finland; 5 Molecular Pathology, University of Tartu, Tartu, Estonia; 6 Centre for Laboratory Medicine, Tampere University Hospital, Tampere, Finland; Centro Cardiologico Monzino IRCCS, Italy

## Abstract

Aging and gender have a strong influence on the functional capacity of the immune system. In general, the immune response in females is stronger than that in males, but there is scant information about the effect of aging on the gender difference in the immune response. To address this question, we performed a transcriptomic analysis of peripheral blood mononuclear cells derived from elderly individuals (nonagenarians, n = 146) and young controls (aged 19–30 years, n = 30). When compared to young controls, we found 339 and 248 genes that were differentially expressed (p<0.05, fold change >1.5 or <−1.5) in nonagenarian females and males, respectively, 180 of these genes were changed in both genders. An analysis of the affected signaling pathways revealed a clear gender bias: there were 48 pathways that were significantly changed in females, while only 29 were changed in males. There were 24 pathways that were shared between both genders. Our results indicate that female nonagenarians have weaker T cell defenses and a more prominent pro-inflammatory response as compared to males. In males significantly fewer pathways were affected, two of which are known to be regulated by estrogen. These data show that the effects of aging on the human immune system are significantly different in males and females.

## Introduction

Old age is associated with a higher risk of inflammatory diseases, autoimmune disorders and malignancies. This increased risk is due to the decreased function of the immune system, with immunosenescence and chronic low-grade inflammation, termed inflamm-aging, representing the key changes [1]. With advancing age, the number of naïve CD4+ and CD8+ T cells declines, while the number of memory and effector cells increases. One prominent feature of old age is the increased proportion of late-stage differentiated CD8+ T cell clones that lack the expression of the costimulatory molecule CD28. Additionally, T cell function is modulated with advancing age; older individuals show a restricted T cell receptor (TCR) repertoire and defects in TCR-mediated signaling [2]. Similar to T cells, the number of naïve B cells is decreased, while the number of memory B cells is increased [3]. Inflamm-aging is another hallmark of aging. In the elderly, the blood levels of pro-inflammatory cytokines (IL-6, TNF-α and CRP) are increased, but the cellular sources and inductive signals underlying this expression are still largely unknown [4].

The immune system shows strong sexual dimorphism. Generally, females are more immunocompetent, meaning that they show increased resilience to various infections and some non-infectious diseases, such as cancer [5]. However, as a result, females are more prone to autoimmune disorders. Sex hormones are correlated with some of these differences, but other physical, and possibly social, factors may have a role in the sexual dimorphism of immune functions [5], [6]. In general, females and males age differently, as most clearly observed in the variance of morbidity and mortality rates between the genders [7]. However, the combined effects of aging and gender on the human immune system have not been analyzed previously.

## Results

**Figure 1:**
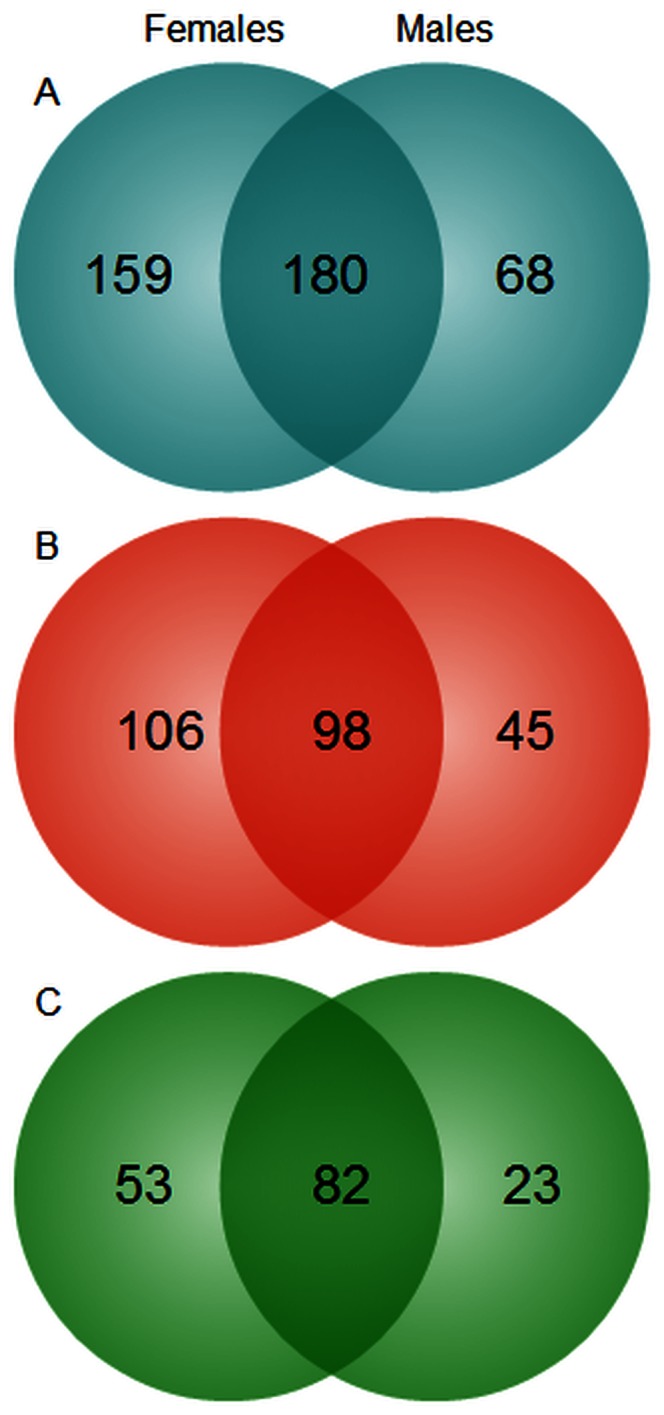
Genes differentially expressed in nonagenarians. We found 339 genes that were differentially expressed in female nonagenarians, compared to young female controls, and 248 genes that were differentially expressed in male nonagenarians, compared to young male controls (p<0.05, −1.5> FC >1.5). A total of 180 of these genes were common to both genders. Slightly more genes were up-regulated (1b) than were down-regulated (1c) in the nonagenarians of both genders.

To better understand the combinatorial effects of age and gender on the immune system, we analyzed the global gene expression profile of peripheral blood mononuclear cells (PBMCs) from nonagenarians (n = 146, 103 females, 43 males) and young controls (n = 30, aged 19–30 years, 21 females, 9 males) using an Illumina Human HT12v4 BeadChip array. The data were analyzed with the Chipster program [8] (IT Center for Science Ltd (CSC), Espoo, Finland). Using a cut-off of p<0.05 and a fold change (FC) of below −1.5 or above 1.5, we identified 339 genes that were differentially expressed in female nonagenarians compared to female controls, and 248 genes that were differentially expressed in male nonagenarians compared to male controls. Of these genes, 180 were common to both genders (Figure 1). The top 10 up- and down-regulated genes are shown in Table 1, and all differentially expressed genes are listed in Tables S1 and S2. The expression levels of four transcripts were verified with qPCR. The transcripts verified included both up- and down-regulated transcript as well as transcripts with high and low FC. The results acquired through qPCR were positively correlated with the microarray results. The expression of CD83, IL8 and LRRN3 were measured, with FCs (microarray/qPCR) in males of 1.73/1.90, 3.46/7.26 and −4.68/−5.65, respectively. In females, the fold changes (microarray/qPCR) for CD83, IL8, LRRN3 and PLCG1 were 1.70/1.71, 4.85/6.15, −5.64/−7.81 and −1.63/−1.98, respectively.

**Table 1:** Ten most up- and down-regulated genes found in females and males.

Gender	Symbol	Description	p	FC
Female	LRRN3*	leucine rich repeat neuronal 3	<0.000001	−5.639
	CCR7	chemokine (C-C motif) receptor 7	<0.000001	−3.613
	LOC652694	similar to Ig kappa chain V-I region HK102 precursor	<0.000001	−2.877
	IGJ	immunoglobulin J polypeptide, linker protein for immunoglobulin alpha and mu polypeptides	<0.000001	−2.848
	CD27	CD27 molecule	<0.000001	−2.747
	CD79A	CD79a molecule, immunoglobulin-associated alpha	<0.000001	−2.680
	CD19	CD19 molecule	<0.000001	−2.651
	IGLL1	immunoglobulin lambda-like polypeptide 1	<0.000001	−2.643
	SGK223	homolog of rat pragma of Rnd2	<0.000001	−2.643
	FCRLA	Fc receptor-like A	<0.000001	−2.628
	IL8*	interleukin 8	<0.000001	4.851
	PTGS2	prostaglandin-endoperoxide synthase 2 (prostaglandin G/H synthase and cyclooxygenase)	<0.000001	3.792
	NR4A2	nuclear receptor subfamily 4, group A, member 2	<0.000001	3.169
	RHOB	ras homolog gene family, member B	<0.000001	2.951
	CDKN1A	cyclin-dependent kinase inhibitor 1A (p21, Cip1)	<0.000001	2.909
	IL1B	interleukin 1, beta	<0.000001	2.840
	RGS1	regulator of G-protein signaling 1	<0.000001	2.766
	EGR1	early growth response 1	<0.000001	2.609
	CCL3L3	chemokine (C-C motif) ligand 3-like 3	<0.000001	2.600
	HBEGF	heparin-binding EGF-like growth factor	<0.000001	2.557
Male	LRRN3*	leucine rich repeat neuronal 3	<0.000001	−4.678
	CD79A	CD79a molecule, immunoglobulin-associated alpha	0.000034	−3.073
	CCR7	chemokine (C-C motif) receptor 7	<0.000001	−3.045
	CD19	CD19 molecule	0.000005	−2.963
	FCRLA	Fc receptor-like A	0.000010	−2.874
	CD79B*	CD79b molecule, immunoglobulin-associated beta	<0.000001	−2.713
	NELL2	NEL-like 2 (chicken)	<0.000001	−2.709
	LOC652694	similar to Ig kappa chain V-I region HK102 precursor	0.000169	−2.423
	LEF1	lymphoid enhancer-binding factor 1	<0.000001	−2.414
	VPREB3	pre-B lymphocyte 3	<0.000001	−2.413
	IL8	interleukin 8	0.007899	3.459
	NR4A2	nuclear receptor subfamily 4, group A, member 2	0.000001	3.422
	JUN	jun proto-oncogene	0.005935	3.046
	RGS1	regulator of G-protein signaling 1	0.000015	2.935
	CDKN1A	cyclin-dependent kinase inhibitor 1A (p21, Cip1)	<0.000001	2.868
	OSM	oncostatin M	0.000784	2.716
	PTGS2	prostaglandin-endoperoxide synthase 2 (prostaglandin G/H synthase and cyclooxygenase)	0.003393	2.684
	HBEGF	heparin-binding EGF-like growth factor	0.000422	2.615
	IL1B	interleukin 1, beta	0.005170	2.561
	ADM	adrenomedullin	0.000034	2.433

The 10 most down- and up-regulated transcripts in female and male nonagenarians compared to young controls. All differentially expressed transcripts are listed in supplementary Tables S1 (males) and S2 (females).

* There are several transcripts of this gene, only the one with largest FC is shown.

To identify the biological pathways affected, Ingenuity Pathway Analysis software (IPA) (Ingenuity® Systems, www.ingenuity.com) was used. Of the pathways by the Ingenuity Knowledge Base, our analysis revealed 48 pathways that were significantly affected in females (p<0.05, FDR<0.25 and at least 3 genes from the pathway were up- or down-regulated) and 29 pathways that were affected in males. Of these pathways, 24 were common to both genders. *B cell development* was the most significantly affected pathway in both genders. Other pathways that were significantly affected included the *Dendritic cell maturation* pathway and *T helper cell differentiation* pathway (Table 2, Table S3). Furthermore, changes in a significant number of pathways were found to be age-dependent in only one of the genders. In females, there were 24 gender-specific pathways (i.e. pathways that were only affected in females), and the most significantly affected signaling pathway was *CTLA4 signaling in cytotoxic T lymphocytes*. In males, there were fewer gender-specific pathways (5 in total), and the *Estrogen mediated S-phase entry* pathway was most affected (Tables 3 and 4). The proportions of different T cell subpopulations in the study subjects were determined with FACS analysis (Table S4) and no statistically significant differences were found between the genders. Thus, unequal representation of different T cell subsets can be excluded as an explanation for these gender-specific differences.

**Table 2:** The canonical pathways that were most affected in nonagenarians of both genders.

Canonical pathway		−logFDR	−logP	Ratio	Rank	Molecules
B Cell Development	Females	6.04	8.40	0.276	1	PTPRC↑, CD19↓, HLA-DOA↓, CD79B↓, HLA-DQA1↑, CD86↑, HLA-DOB↓, CD79A↓
	Males	5.43	7.86	0.241	1	PTPRC↑, CD19↓, HLA-DOA↓, SPN↑, CD79B↓,HLA-DOB↓,CD79A↓
B Cell Receptor Signaling	Females	2.04	3.29	0.062	12	PTPRC↑, CD19↓, JUN↑, CD79B↓, FCGR2A↑, EGR1↑, PIK3AP1↑, CREB5↑, BCL6↑, CD79A↓
	Males	1.06	2.32	0.043	11	PTPRC↑, CD19↓, JUN↑, CD79B↓, EGR1↑, BCL6↑, CD79A↓
Communication between Innateand Adaptive Immune Cells	Females	3.48	5.15	0.097	4	IL8↑, TLR10↓, IL15↑, CCL3L1/CCL3L3↑, CD86↑, IL1B↑, CD83↑, CCL3↑, CCR7↓
	Males	1.75	3.34	0.065	5	IL8↑, TLR10↓, CCL3L1/CCL3L3↑, IL1B↑, CD83↑, CCR7↓
Crosstalk between Dendritic Cells and Natural Killer Cells	Females	2.36	3.72	0.089	9	LTA↓, CD69↑, IL15↑, LTB↓, CD86↑, CD83↑, CCR7↓, FAS↑
	Males	0.62	1.52	0.044	21	KIR3DL2↑, LTB↓, CD83↑, CCR7↓
Dendritic Cell Maturation	Females	6.04	8.24	0.089	2	HLA-DOA↓, LEP↑, FCGR2A↑, IL15↑, HLA-DQA1↑, PLCG1↓, LTB↓, CD83↑, CREB5↑, FCGR1A↑, LTA↓, HLA-DOB↓, CD86↑, IL1B↑, STAT1↑, CCR7↓, FCGR1B↑
	Males	1.84	3.49	0.047	4	HLA-DOA↓, LTB↓, HLA-DOB↓, IL1B↑, CD83↑, STAT1↑, FCGR1A↑, CCR7↓, FCGR1B↑
IL-8 Signaling	Females	2.48	3.90	0.063	8	IL8↑, GNG11↑, JUN↑, RHOB↑, DEFA1↑, VEGFB↓, HBEGF↑, PTGS2↑, IRAK3↑, ITGB5↑, MMP9↑, GNG7↓
	Males	0.62	1.44	0.031	26	IL8↑, JUN↑, RHOB↑, HBEGF↑, PTGS2↑, GNG7↓
ILK Signaling	Females	2.07	3.34	0.060	11	MYC↓, FLNB↓, JUN↑, RHOB↑, VEGFB↓, LEF1↓, HIF1A↑, PTGS2↑, CREB5↑, ITGB5↑, MMP9↑
	Males	0.62	1.46	0.032	25	MYC↓, JUN↑, RHOB↑, LEF1↓, HIF1A↑, PTGS2↑
Oncostatin M Signaling	Females	1.47	2.50	0.118	21	MT2A↑, IL6ST↓, OSM↑, STAT1↑
	Males	1.45	2.98	0.118	6	MT2A↑, IL6ST↓, OSM↑, STAT1↑
OX40 Signaling Pathway	Females	1.70	2.77	0.082	19	HLA-DOA↓, JUN↑, HLA-DQA1↑, HLA-DOB↓, TRAF5↓
	Males	1.11	2.42	0.066	9	HLA-DOA↓, JUN↑, HLA-DOB↓, TRAF5↓
PI3K Signaling in B Lymphocytes	Females	2.64	4.11	0.078	7	BLK↓, PTPRC↑, CD19↓, IL4R↓, JUN↑, ATF3↑, CD79B↓, PLCG1↓, PIK3AP1↑, CD79A↓
	Males	1.45	2.89	0.055	7	BLK↓, PTPRC↑, CD19↓, JUN↑, ATF3↑, CD79B↓, CD79A↓
Role of JAK family kinases in IL-6-type Cytokine Signaling	Females	1.87	3.01	0.154	16	IL6ST↓, SOCS3↑, OSM↑, STAT1↑
	Males	1.84	3.51	0.154	3	IL6ST↓, SOCS3↑, OSM↑, STAT1↑
Role of NFAT in Regulation of the Immune Response	Females	3.48	5.14	0.070	5	HLA-DOA↓, CD79B↓, FCGR2A↑, HLA-DQA1↑, PLCG1↓, FCGR1A↑, GNG7↓, CD79A↓, GNG11↑, JUN↑, CD86↑, HLA-DOB↓, FCGR1B↑
	Males	1.45	2.84	0.043	8	HLA-DOA↓, JUN↑, CD79B↓, HLA-DOB↓, FCGR1A↑, GNG7↓, CD79A↓, FCGR1B↑
T Helper Cell Differentiation	Females	4.00	5.80	0.130	3	IL6ST↓, IL4R↓, HLA-DOA↓, HLA-DQA1↑, CD86↑, HLA-DOB↓, CXCR5↓, STAT1↑, BCL6↑
	Males	1.93	3.76	0.087	2	IL6ST↓, HLA-DOA↓, HLA-DOB↓, CXCR5↓, STAT1↑, BCL6↑
TREM1 Signaling	Females	2.70	4.25	0.123	6	IL8↑, TLR10↓, PLCG1↓, CD86↑, IL1B↑, CD83↑, CCL3↑
	Males	1.01	2.21	0.070	12	IL8↑, TLR10↓, IL1B↑, CD83↑

P-values were derived from Fisher’s exact test, -log(0.05) = 1.3, and the Benjamini-Hochberg-corrected t-test (FDR), –log(0.25) = 0.61. The ratio is the number of differentially expressed genes in the data set divided by the total number of genes in the given pathway. The rank indicates the position of the pathway in the gender-specific pathway list. A complete list of the pathways affected in both genders is shown in supplementary Table S3 .

**Table 3:** The canonical pathways that were affected in nonagenarian females.

Canonical pathways	−logFDR	−logP	Ratio	Molecules
Prostanoid Biosynthesis	2.07	3.34	0.333	PTGS1↑, PTGS2↑, PTGDS↑
CTLA4 Signaling in Cytotoxic T Lymphocytes	1.90	3.06	0.074	HLA-DOA↓, HLA-DQA1↑, TRAT1↓, PLCG1↓, CD86↑, HLA-DOB↓, CTLA4↑
CCR5 Signaling in Macrophages	1.90	3.06	0.074	GNG11↑, JUN↑, PLCG1↓, CCL3↑, GNG7↓, FAS↑
IL-15 Production	1.77	2.88	0.138	TXK↓, IL15↑, STAT1↑, IRF1↑
IL-10 Signaling	1.76	2.85	0.083	IL1R2↑, SOCS3↑, IL4R↓, JUN↑, FCGR2A↑, IL1B↑
p38 MAPK Signaling	1.36	2.33	0.060	IL1R2↑, MYC↓, IL1B↑, IRAK3↑, CREB5↑, STAT1↑, FAS↑
P2Y Purigenic Receptor Signaling Pathway	1.30	2.25	0.056	MYC↓, ITGA2B↑, GNG11↑, JUN↑, PLCG1↓, CREB5↑, GNG7↓
iNOS Signaling	1.26	2.17	0.087	JUN↑, IRAK3↑, STAT1↑, IRF1↑
Cytotoxic T Lymphocyte-mediated Apoptosis ofTarget Cells	1.24	2.13	0.077	HLA-DOA↓, HLA-DQA1↑, HLA-DOB↓, FAS↑
Differential Regulation of Cytokine Production in Intestinal Epithelial Cells by IL-17A and IL-17F	1.23	2.10	0.130	LCN2↑, IL1B↑, CCL3↑
iCOS-iCOSL Signaling in T Helper Cells	1.21	2.06	0.054	PTPRC↑, HLA-DOA↓, HLA-DQA1↑, TRAT1↓, PLCG1↓, HLA-DOB↓
IL-4 Signaling	1.19	2.04	0.067	IL4R↓, HLA-DOA↓, HLA-DQA1↑, HLA-DOB↓, FCER2↓
Nur77 Signaling in T Lymphocytes	1.19	2.03	0.070	HLA-DOA↓, HLA-DQA1↑, CD86↑, HLA-DOB↓
PKCθ Signaling in T Lymphocytes	1.06	1.87	0.047	HLA-DOA↓, JUN↑, HLA-DQA1↑, PLCG1↓, CD86↑, HLA-DOB↓
TNFR2 Signaling	1.06	1.86	0.094	JUN↑, LTA↓, TNFAIP3↑
Calcium-induced T Lymphocyte Apoptosis	1.02	1.82	0.066	HLA-DOA↓, HLA-DQA1↑, PLCG1↓, HLA-DOB↓
G Protein Signaling Mediated by Tubby	0.97	1.74	0.081	GNG11↑, PLCG1↓, GNG7↓
Glucocorticoid Receptor Signaling	0.97	1.74	0.036	IL1R2↑, IL8↑, JUN↑, SGK1↑, CDKN1A↑, IL1B↑, PTGS2↑, STAT1↑,CCL3↑, FCGR1A↑
Inhibition of Angiogenesis by TSP1	0.93	1.67	0.091	JUN↑, THBS1↑, MMP9↑
Role of JAK1 and JAK3 in γc Cytokine Signaling	0.91	1.64	0.064	SOCS3↑, IL4R↓, IL15↑, STAT1↑
ERK5 Signaling	0.87	1.59	0.063	IL6ST↓, MYC↓, SGK1↑, CREB5↑
Antigen Presentation Pathway	0.86	1.57	0.075	HLA-DOA↓, HLA-DQA1↑, HLA-DOB↓
Production of Nitric Oxide and Reactive OxygenSpecies in Macrophages	0.68	1.35	0.038	JUN↑, RHOB↑, PLCG1↓, PCYOX1↑, STAT1↑, SPI1↑, IRF1↑
Phospholipase C Signaling	0.66	1.33	0.033	GNG11↑, CD79B↓, RHOB↑, FCGR2A↑, PLCG1↓, CREB5↑, GNG7↓, CD79A↓

The pathways that were significant only in females are shown. The p-values were derived from Fisher’s exact test, −log(0.05) = 1.3 and the Benjamini-Hochberg-corrected t-test (FDR), −log(0.25) = 0.61. The ratio is the number of differentially expressed genes in the data set divided by the total number of genes in the given pathway.

**Table 4:** The canonical pathways that were affected in nonagenarian males.

Canonical pathways	−logFDR	−logP	Ratio	Molecules
Estrogen-mediated S-phase Entry	1.11	2.41	0.111	MYC↓, CDKN1A↑, E2F5↓
PDGF Signaling	0.77	1.71	0.051	MYC↓, JUN↑, STAT1↑, PDGFRB↑
CD27 Signaling in Lymphocytes	0.62	1.52	0.055	JUN↑, TRAF5↓, CD27↓
PPAR Signaling	0.62	1.49	0.040	JUN↑, IL1B↑, PTGS2↑, PDGFRB↑
Role of Pattern Recognition Receptors in Recognitionof Bacteria and Viruses	0.62	1.48	0.042	NLRP3↑, C5AR1↑, IL1B↑, OAS3↑

The pathways that were significant only in males are shown. The p-values were derived from Fisher’s exact test, −log(0.05) = 1.3 and the Benjamini-Hochberg-corrected t-test (FDR), −log(0.25) = 0.61. The ratio is the number of differentially expressed genes in the data set divided by the total number of genes in the given pathway.

## Discussion

In summary, the data presented here suggests that the effect of aging on the function of the human immune system is different between males and females. Aging-associated changes in gene expression have previously been studied in PBMCs [9], [10], but these studies did not consider the effect of gender. Several genes identified in our study have previously been associated with aging or advanced age, including LEF1 [9], [10], [11], [12], VPREB3 [10], NR4A2 [11], LRRN3 [9], [10], [12], CCR7 [12], [13] and CD19 [13]. All of these genes were affected in both genders. All of the pathways that were found to be significantly affected by aging in both genders of nonagenarians in this study have been reported associated with aging in the literature. We also identified one novel pathway, *TREM1 signaling*, that has not been previously associated with aging. *TREM1 signaling* has a role in acute inflammation; it is expressed in blood neutrophils and monocytes, and its expression is induced by pathogens (LPS, bacteria and fungi [14], [15]). It appears that the *TREM1 signaling* pathway contributes to the pro-inflammatory state in elderly populations.

In addition to the pro-inflammatory pathways that are affected in nonagenarians of both genders, several pro-inflammatory pathways were affected only in females (Table 3). This result is not surprising, because females generally to have stronger inflammatory reactions [6]. One reason for the muted inflammatory response in males may be testosterone, which is known to have anti-inflammatory effects [16]. NF-κB signaling is affected in both genders, but in females, there were more genes with this pathway that were affected. In addition, two NO synthesis-associated pathways were affected only in females, which indicates more potent NF-κB signaling and an elevated pro-inflammatory response in females because iNOS induction and NO synthesis are induced by NF-κB and other pro-inflammatory cytokines [17]. The *p38 MAPK signaling* pathway, which was significantly affected in females only, can also be activated by cell stressors other than pro-inflammatory cytokines [18]. This result indicates that females may have a more potent stress response than males.

Several of the age dependent, female-specific pathways are involved in the activation of T lymphocytes (Table 3), suggesting that gender may play an important regulatory role in T cell-mediated defense. Because the expression levels of several genes in these pathways were either up- or down regulated, it is difficult to reconstruct the functional end result. However, because the *CTLA4 signaling in cytotoxic T lymphocytes* pathway, including elevated expression of CTLA4, and the *iCOS-iCOSL signaling in T helper cells* pathway were significantly affected in females only, it appears that the T cell-mediated defense is weaker in female nonagenarians than in male nonagenarians.

The *Cytotoxic T lymphocyte mediated apoptosis of target cells* pathway was also found to be affected by age in females only. Chronic viral infections, e.g. cytomegalovirus (CMV) and Epstein-Barr virus, affect a large majority of the nonagenarian population. In our nonagenarian study population 96% of the females and 95% of the males were seropositive for CMV. Cytotoxic CD8+ cells are the primary cell type that controls these viral infections, but chronic viral infections can also induce the generation of atypical cytotoxic CD4+ cells, which express granzyme B [19]. Because chronic viral infections are thought to be a driving force behind age-associated changes in the immune system, and because there is no difference in seroprevalence between the genders, it is of great interest to determine whether the immune systems of males and females control these infections in different ways. Additionally, the production of IL-15, a major homeostatic cytokine, was affected only in females (the *IL-15 production* pathway). Previously, it was shown that the blood levels of this cytokine are elevated in centenarians [20], [21], but our data show that this phenomenon is restricted to female nonagenarians, at least at the transcriptional level (FC = 1.6).

Significantly fewer pathways were affected in males than in females (Table 4). Males also had fewer differentially expressed transcripts, but this does not completely explain the difference in the number of pathways, as the canonical pathway analysis in IPA takes into account the number of input transcripts. The *Estrogen mediated S-phase entry* pathway was most significantly changed. Generally, estrogen is known to have an effect on inflammation, and to possibly have a protective role against oxidative stress. The levels of estrogen and androgens decrease in aging males and low levels of estrogen are associated with a risk of fracture. However, the relative contribution of estrogens versus androgens in aging males is unclear [16]. *PDGF signaling*, which was also affected in males, has also been shown to be affected by a lack of estrogen [22].

The study presented here has some limitations. We have shown that the proportions of different T cell subpopulations or the proportion of monocyte-macrophage lineage cells do not differ between the genders (Table S4). However, there may be differences in the proportion of other cell populations that may explain some of the observed differences in gene expression.

The number of healthy young controls used is relatively small in comparison to nonagenarian group. Thus, the small sample size will have an effect on the power of statistical testing to identify differentially expressed genes. To address this limitation, we have used statistical test specifically designed for small sample sizes. Data interpretation through pathway enrichment also mitigates this limitation as we do not need to observe all, only a significant fraction of genes belonging to a given pathway.

Additionally, we have previously shown that aging-related changes are affected by the CMV serostatus [23]. Because of the high seroprevalence of CMV in the nonagenarian study population (96% of females and 95% of males are seropositive for CMV), we cannot assess the combinatorial effect of gender and CMV on the age-associated changes in transcription.

This study focused on the effect of aging on the immune systems of males and females. It has been known for decades that gender has an influence on the function of the immune system, with females generally having a stronger immune response. Gender differences in the immune response are also detectable at transcriptomic levels [24]. Sex steroids, estrogen and testosterone, clearly play a role in driving gender differences in the immune response. Presently, there is no biological explanation for these aging-induced differences, and we can only speculate based on the available evidence. For example, aging strongly influences the levels of sex steroids, but during menopause estrogen levels decrease more rapidly than testosterone levels do during andropause [16]. Specifically, the positive effects of estrogens on the immune system stop at about age 45–55.

Another interesting possibility involves potential changes in the X-chromosome. The X-chromosome contains the largest number of immune-related genes in the genome [25], and aging may modify the function of genes on the X-chromosome. In females, X-chromosome is inactivated at random during an early embryonic stage (i.e. there is a 50/50 ratio of the maternal and paternal X-chromosomes). However, in elderly individuals, this ratio may be skewed. During a 13-year follow-up it was recently shown that this skewing is associated with survival [26]. It remains to be established whether this skewing has an influence on the expression of immune-related genes.

## Materials and Methods

### Ethics Statement

All participants in this study provided their written, informed consent. This study was conducted according to the principles expressed in the declaration of Helsinki, and the study protocol was approved by the ethics committee of the city of Tampere (Study protocol number SOTE 1592/402/96).

### Population

The study population consisted of 146 nonagenarians (females n = 103, males n = 43) who were participating in the Vitality 90+ study, and 30 young, healthy controls (aged 19–30 years, median 22.5 years; females n = 21, males n = 9). All of the study subjects were of Western European descent. The Vitality 90+ study is an ongoing prospective population-based study that includes both home-dwelling and institutionalized individuals aged 90 years or more who live in the city of Tampere, Finland. The recruitment and characterization of the participants were performed as previously reported for earlier Vitality 90+ study cohorts [27]. In this study, we included only individuals born in 1920, and the samples used in this study were collected in the year 2010. The nonagenarians included in the study had not had any infections or received any vaccinations in the 30 days prior to the blood sample collection. The young controls consisted of healthy laboratory personnel who did not have any medically diagnosed chronic illnesses, who were non-smokers and who had not had any infections or received any vaccinations within the two weeks prior to the blood sample collection.

### Sample Collection

The blood samples were collected into EDTA-containing tubes by a trained medical student during a home visit. All of the blood samples were drawn between 8 am and 12 am. The samples were directly subjected to leucocyte separation with a Ficoll-Paque density gradient (Ficoll-Paque™ Premium, cat.no. 17-5442-03, GE Healthcare Bio-Sciences AB, Uppsala, Sweden). The PBMC layer was collected and a subset of cells was suspended in 150 µl of RNAlater solution (Ambion Inc., Austin, TX, USA) for use in a microarray analysis. The cells that were to be used for FACS analysis were suspended in 1 ml of a Freezing solution (5/8 FBS, 2/8 RPMI-160 medium, 1/8 DMSO) (FBS cat. no. F7524, Sigma-Aldrich, MO, USA; RPMI: cat. no. R0883, Sigma-Aldrich, MO, USA; DMSO: cat. no. 1.02931.0500, VWR, Espoo, Finland).

### RNA Extraction and Transcriptomic Analysis

For RNA extraction, equal amounts of PBS and RNAlater were added to the cell suspension and then removed by centrifugation, leaving only the cell pellet. RNA was purified using an miRNeasy mini kit (Qiagen, CA, USA) according to the manufacturer’s protocol using on-column DNase digestion (AppliChem GmbH, Darmstadt, Germany). The concentration and quality of the RNA were assessed with an Agilent RNA 6000 Nano Kit on Agilent 2100 Bioanalyzer (Agilent Technologies, CA, USA).

Labeled cRNA was prepared from 330 ng of total RNA using an Illumina TotalPrep RNA Amplification Kit (Ambion Inc., TX, USA) with overnight incubation according to the manufacturer’s protocol. The quality of the labeled cRNA was determined using a 2100 Bioanalyzer (Agilent Technologies). In total, 1,500 ng of labeled cRNA was hybridized to a HumanHT-12 v4 Expression BeadChip (Cat no. BD-103-0204, Illumina Inc., CA, USA) overnight according to the Illumina protocol in the Core Facility at the Department of Biotechnology, University of Tartu. The chips were scanned using a Beadscan (Illumina Inc.). The microarray data are available in the GEO database (http://www.ncbi.nlm.nih.gov/geo/), accession number GSE40366.

### Data Preprocessing and Statistical Analysis

The preprocessing, filtering and analysis of the data were performed with the Chipster v2.3 program [8] (CSC, Espoo, Finland). The lumi pipeline was used for data preprocessing and normalization [28]. The Array_Address_ID was used as a probe identifier, background correction was performed with the bgAdjust.affy package, and the data were transformed with the vst (variance stabilizing transformation) method and normalized with the rsn (robust spline normalization) method. The vst and rsn methods were chosen, because they are recommended in the literature and are designed to take in to account the technical replicates in each Illumina chip (the bead array technology) [28], [29], [30]. The quality control was performed by using box blot, density blot and PCA analysis.

To filter out the non-expressed probes and probes whose expression did not change between study groups, we filtered the data based on the coefficient of variation (CV, standard deviation/mean). We included the 5% of probes (2367) with the highest CV, i.e., the highest variation between nonagenarian and control samples. The nonagenarian samples and control samples were compared with an empirical Bayes two-group test from the limma package [31] using the Benjamini-Hochberg false discovery rate (FDR) for multiple testing correction. The threshold for significance for p-values was set to 0.05. From these genes, we classified those with a linear fold change above 1.5 or below -1.5 as differentially expressed. This classification was performed to obtain comparable groups of genes from both genders. Because more females were included in the study, performing the analysis without a fold change limit produced almost three times more genes for the females compared with the males.

### IPA

To identify canonical pathways associated with aging, we analyzed the gene sets with IPA software (Ingenuity® Systems, www.ingenuity.com). According to the manufacturer, the canonical pathways are well-characterized metabolic and cell signaling pathways that have been curated and hand-drawn by PhD-level scientists. The information used to construct the canonical pathways is derived from specific journal articles, review articles, text books, and the KEGG Ligand database. The canonical pathways are directional. All of the data sources provided by the Ingenuity Knowledge Base were included in the IPA analysis. For the association of molecules, only experimentally observed results were accepted and only human data were considered. The HumanHT-12 v 4.0 was used as a reference set to generate p-values for the pathways, and Fisher’s exact test and Benjamini-Hochberg multiple testing correction (FDR) were used to calculate p-values for the pathways. With these parameters, we obtained 293 and 213 analysis-ready molecules for females and males, respectively, out of a total of 339 and 248 genes that were differentially expressed. We considered a canonical pathway to be significantly affected at p<0.05 (-logP>1.3), at FDR<0.25 and when the pathway contained a minimum of 3 genes. Pathways associated with cancer and other disease, as defined by Ingenuity Systems®, were excluded from the analysis. The IPA analysis was performed on 6.3.2013.

### qPCR Verification

In total, 300 ng of RNA was converted to cDNA using a High Capacity cDNA Reverse Transcription Kit (Part No. 4368814, Applied Biosystems, CA, USA). Because the amount of cDNA was limited, we performed a pre-amplification step using TaqMan® PreAmp Master Mix (Part No. 4348266, Applied Biosystems). This protocol amplifies small amounts of cDNA without introducing bias to the sample. In brief, 15 ng of cDNA was amplified for 10 cycles according to the manufacturer’s instructions using the same assays with which the actual qPCRs were performed (CD83 Hs01077168_g1, IL8 Hs00174103_m1, LRRN3 Hs00539582_s1, PLCG1 Hs01008225_m1 and GUSB Hs00939627_m1 as endogenous control). GUSB was selected as the control gene because GUSB was shown to be the most stable transcript in gene expression studies that used aging PBMCs [32]. The pre-amplified product was diluted 1∶5 with TE- buffer.

Transcript levels were determined with the single gene assays described above, using TaqMan® Gene Expression Master Mix (Part No. 4369016, Applied Biosystems). To determine whether the transcripts were differentially expressed between the nonagenarians and the young controls, we calculated the RQ values with RQ Manager Software (Applied Biosystems).

### FACS

The proportions of different lymphocyte populations were determined using FACS analysis (BD FACSCanto II) and the results were analyzed with BD FACS Diva, version 6.1.3 (BD Biosciences, Franklin Lakes, NJ, USA). The antibodies used were FITC-CD14 (cat. no. 11-0149), PerCP-Cy5.5-CD3 (45-0037), APC-CD28 (17-0289) (eBioscience, San Diego, CA, USA), PE-Cy™7-CD4 (cat. no. 557852) and APC-Cy™7-CD8 (557834) (BD Biosciences).

## Supporting Information

Table S1 **Differentially expressed transcripts in male nonagenarians compared to male controls.**
(XLSX)

Table S2 **Differentially expressed transcripts in female nonagenarians compared to female controls.**
(XLSX)

Table S3 **Affected canonical pathways in both genders.**
(XLSX)

Table S4 **Proportions of T cell populations in nonagenarians and young controls of both genders.**
(XLSX)
